# Effect of Preheating Whey Protein Concentrate on the Stability of Purple Sweet Potato Anthocyanins

**DOI:** 10.3390/polym15153315

**Published:** 2023-08-06

**Authors:** Shuo Zhang, Guowei Deng, Fang Wang, Haiyan Xu, Jiagen Li, Jialei Liu, Dengfeng Wu, Shitao Lan

**Affiliations:** 1 Sichuan Provincial Key Laboratory for Development and Utilization of Characteristic Horticultural Biological Resources, College of Chemistry and Life Sciences, Chengdu Normal University, Chengdu 611130, China; 15388114168@163.com (S.Z.); guoweideng@cdnu.edu.cn (G.D.); lijiagen0204@outlook.com (J.L.); wudengfeng0804@163.com (D.W.); lanshitao2002@163.com (S.L.); 2 College of Life Sciences, Sichuan Normal University, Chengdu 610101, China; weiliangxhy@163.com; 3 Institute of Environment and Sustainable Development in Agriculture, Chinese Academy of Agricultural Sciences, Beijing 100081, China

**Keywords:** preheating temperature, preheating time, whey protein concentrate, anthocyanins, stability, interaction

## Abstract

Anthocyanins (ANs) have strong antioxidant activities and can inhibit chronic diseases, but the instability of ANs limits their applications. The conservation of preheating whey protein concentrate (WPC) on the stability of purple sweet potato ANs was investigated. The retention of ANs in WPC-ANs was 85.88% after storage at 25 °C for 5 h. WPC-ANs had higher retention of ANs in heating treatment. The retention rates of ANs in WPC-ANs exposed to light and UV lamps for 6 h were 78.72% and 85.76%, respectively. When the concentration of H_2_O_2_ was 0.50%, the retention rate of ANs in the complexes was 62.04%. WPC-ANs’ stability and antioxidant activity were improved in simulated digestive juice. The WPC-ANs connection was static quenching, and the binding force between them was a hydrophobic interaction at one binding site, according to the fluorescence quenching spectroscopy. UV-visible absorption spectroscopy and Fourier transform infrared spectroscopy (FTIR) analysis further indicated that the secondary structure and microenvironment of amino acid residues in WPC can be impacted by the preheating temperature and preheating times of WPC. In conclusion, preheating WPC can successfully preserve the stability of purple sweet potato ANs by binding to them through a non-covalent interaction.

## 1. Introduction

An essential component for the food business, anthocyanins (ANs) are found in abundance in purple sweet potatoes [1,2]. ANs are natural colorants that confer blue, purple, and red color to vegetables and fruits [3]. The flavylium cation (2-phenylbenzopyrylium) is the primary structural component of anthocyanins. It is a C_6_-C_3_-C_6_ structure composed of an aromatic ring attached to a heterocyclic pyran ring through a carbon-carbon bond [4]. The quantity and placement of hydroxyl and methoxyl groups, the type and placement of the sugars attached to the flavylium ring, and the quantity and nature of the aliphatic or aromatic acids related to the sugar groups all varied between ANs structures [5]. Six primary ANs aglycones have been found in nature, according to diverse substituent patterns on various places on the B rings: pelargonidin (Pg), petunidin (Pt), peonidin (Pn), malvidin (Mv), cyanidin (Cy), and delphinidin (Dp) [6]. ANs have strong anti-oxidant [7] and anti-cancer activities [8]. They also have activities in inhibiting chronic diseases, like diabetes [9], and obesity [10]. However, the instability of ANs limits the range of applications. They are easily influenced by pH, temperature, light, pressure, oxygen, enzymes, and ascorbic acid [11]. For this reason, figuring out how to increase ANs stability is essential for using it in food [12].

By interacting with natural whey protein (WP) and forming chemical complexes, ANs’ stability can be improved. WP is an important component of milk protein, which mainly includes β-lactoglobulin (β-Lg), α-lactalbumin (α-La), bovine serum albumin (BSA), and immunoglobulin (IgG) [13]. Through non-covalent and covalent interactions, WP and ANs can create composite materials. Hydrophobic interactions, hydrogen bonding, electrostatic attraction, and van der Waals forces (VDW) all contribute to the non-covalent interactions. Non-covalent interactions between proteins and ANs are weak but common in food. They all have the potential to cause major changes in the structure, function, and nutrition of individual molecules to varying degrees and serve to protect anthocyanin stability [14,15]. ANs and proteins exhibit a variety of binding behaviors as a result of structural variations. Blueberry anthocyanins and WP interact through hydrophobic interactions [16]. Anthocyanins from red raspberry pomace extract interact with WP through electrostatic interactions [17]. VDW mediates the interaction between purple potato anthocyanins and WP [18]. The interaction of purple sweet potato ANs with whey protein concentrate (WPC) has not been investigated.

The interaction between WP and ANs is facilitated by heat treatment. Milk proteins’ secondary and tertiary structures are altered by heat treatment, which affects the proteins’ ability to bind to other molecules. Preheating WP has a better protective impact than native WP on the thermal, oxidation, and photo stability of grape skin ANs extracts, according to a prior study [19]. Ren et al. (2022) used FTIR to investigate the WP-ANs interaction. The WP structure became more disordered at higher preheating temperatures (70–80 °C), which introduced a stronger WP-ANs interactions owing to amide III alterations [20]. However, information on how preheating conditions affect WPC-ANs interactions is still lacking.

This study sought to understand how preheated WPC and purple sweet potato anthocyanins interact with each other. We evaluated the WPC-ANs complex’s stability and antioxidant activity under various processing scenarios. Fluorescence spectroscopy was utilized to investigate the binding pattern of WPC to ANs. The effects of preheating temperatures (25 °C, 60 °C, 70 °C, 80 °C, and 90 °C) and preheating times (0, 15, 30, 45, 60, and 75 min) on the secondary structure of WPC were examined using UV-visible absorption spectroscopy and FTIR. This systematic investigation of the interactions between the preheated WPC and ANs could benefit the extraction and utilization of purple sweet potato anthocyanins. However, the limitations of the study should be recognized. ANs is a common natural colorant sourced from fruits. In this study, the possibility of preheating WPC to prevent ANs color degradation was not investigated.

## 2. Materials and Methods

### 2.1. Materials

Purple sweet potato anthocyanins (25% purity) were acquired from Qufu Shengjiade Biotechnology Co., Ltd. (Qufu, China). Whey protein concentrate (80% purity), DPPH (2,2-diphenyl-1-picrylhydrazyl), amylase, pepsin, and trypsin were acquired from Hefei Bomei Biotechnology Co., Ltd. (Hefei, China). Fluorescein isothiocyanate was acquired from Shanghai Alading Biotechnology Co., Ltd. (Shanghai, China). Other chemical reagents were purchased from Shandong Longkete Enzyme Preparation Co., Ltd. (LIinyi, China) and were of analytical grade.

### 2.2. Heat Treatment of WPC and Preparation of WPC-ANs Complexation

The procedure of He et al. (2016) was followed, with the necessary adjustments, in the manufacture of preheating WPC-ANs [19]. In 0.01 mol/L phosphate buffers (PBS) with a pH of 7.0, WPC (0.5 mg/mL) was prepared. The protein sample was promptly cooled in cold water after being heated at 80 °C for 60 min to prevent any additional denaturation. Anthocyanins at a concentration of 0.5 mg/mL were added to the heated protein solutions. These mixtures are used for stability testing. Different heat treatment temperatures (25, 60, 70, 80, and 90 °C) and times (0, 15, 30, 45, 60, and 75 min) were chosen as factors to evaluate the interaction of preheating WPC-ANs while keeping other conditions constant.

### 2.3. Storage, Thermal, Oxidation, and Photo Stability Testing

The WPC-ANs solution was placed in two tubes and stored at 4 °C and 25 °C for 6 h, protected from light. The remaining WPC-ANs solutions were heated at 60 °C, 70 °C, 80 °C, and 90 °C for 6 h, protected from light. At 1 h intervals, each sample was rapidly chilled, and the ANs content and retention rate were both determined.

The oxidation stability of both the ANs and WPC-ANs samples was evaluated using the Mastufuj et al. (2007) method, with the necessary adjustments [21]. Oxidation stability was tested by adding H_2_O_2_ (0.5%, 1%, or 1.5%), followed by 1 h of darkness at 25 °C. The samples’ ANs content and the retention rate were calculated after oxidation testing.

In separate test tubes, the WPC-ANs solutions were placed for 6 h each in a dark atmosphere, fluorescent light, and UV lamp. In order to figure out their retention rate of ANs, aliquots were taken at 1 h, 2 h, 3 h, 4 h, 5 h, and 6 h. The pH differential method was used to measure the sample’s absorbance at 535 nm and 700 nm [22]. Equation (1) was used to calculate the ANs content.

(1) $$C=\frac{\left(A_{pH1}-A_{pH4.5}\right)\times M_{w}\times DF}{M_{a}\times L}$$

where A is the maximum absorbance; M_w_ is the molecular weight (449,200 mg/mol); DF is the dilution factor; M_a_ is the extinction coefficient (26,900 mol/L ∗ cm); L is the path length (1 cm).

Equation (2) was used to calculate the ANs retention rate, and all samples were compared to the starting content:

(2) $$Retention\;Rate\;(\%)=\frac{Treated\;ANs\;content}{Original\;ANs\;content}\times 100\%$$

### 2.4. The Stability of WPC-ANs during Simulated In Vitro Digestion

Following Brodkorb’s approach, digestion stock solutions (SSF: oral, SGF: gastric, and SIF: intestine) were prepared and kept at −20 °C [23]. The WPC-ANs (pH 7.0) were mixed with SSF, agitated, and then heated to 37 °C in a water bath for 10 min. Oral digestive juices (pH 3.0) were mixed with SGF and agitated in a water bath at 37 °C for two hours. The gastric digestive juices (pH 7.0) were mixed with SIF and agitated in a water bath at 37 °C for two hours. Following each digestion stage, samples were taken and kept in a −80 °C refrigerator. The pH differential method was used to measure the sample’s absorbance at 535 nm and 700 nm. Equation (2) was used to get the ANs retention rate.

### 2.5. Antioxidant Activity Testing

Simulated digestive juice’s reducing power [24], hydroxyl radical scavenging activity [25], 1,1-diphenyl-2-picrylhydrazyl (DPPH) assays [26], metal iron chelating activity [27], and lipid peroxidation inhibition ability [27] had all been investigated.

### 2.6. Fluorescence Spectroscopy

Fluorescence spectroscopy tests were performed on the ANs and WPC-ANs samples using the modified version of Zhang et al.’s methodology [28]. The studies were conducted at three different temperatures: 25, 35, and 45 °C (298, 308, and 318 K). The following settings were made for the fluorescence spectrometer: the excitation wavelength was 280 nm, the emission wavelength range was 300–500 nm, and the slit width was 5.0 nm.

In order to determine *K_SV_*, *K_a_*, and *n*, respectively, the fluorescence intensity was included in the Stern–Volmer Equation (3) and the double logistic regression Equation (4) [16,29].

(3) $$F_{0}/F=1+K_{SV}\left[Q\right]=1+K_{q}\tau_{0}\left[Q\right]$$

where *F*_0_ and *F* are the fluorescence emission intensities of WPC without and with ANs, respectively; [*Q*] is the concentration of the quencher, mmol/L; *K_SV_* is the Stern–Volmer quenching constant, L/mol; *K_q_* is the bimolecular quenching rate constant, L/(mol·s); and *τ*_0_ is the lifetime of the biomolecule without the quencher, (10^−8^ s).

(4) $$\log\left[\left(F_{0}-F\right)/F\right]=\log K_{a}+n\log\left[Q\right]$$

where *K_a_* is the binding constant, L/mol; *n* is the number of binding sites.

Calculation of thermodynamic parameters during the interaction of ANs with WPC according to Van’t Hoff Equations (5) and (6) [16]:

(5) $$∆H=d\left(\frac{∆G}{T}\right)/d\frac{1}{T}$$

(6) $$∆G=-RT\ln K_{a}=∆H-T∆S$$

where ΔH is the change in enthalpy, kJ/mol; ΔG is the change in free energy, kJ/mol; ΔS is the change in entropy, J/mol; R is the gas constant (8.314 J mol^−1^K^−1^); T is the experimental temperature.

### 2.7. UV-Visible Absorption Spectroscopy

The UV-visible absorption spectroscopy of the ANs and WPC-ANs samples was evaluated using a modified version of the Attaribo et al. (2020) method [30]. Samples (10 M) were used to capture UV-visible absorption spectra at 298 K in the 260–480 nm range in a quartz cuvette.

### 2.8. FTIR Spectroscopy

Both the ANs and WPC-ANs samples were subjected to FTIR spectroscopy testing in accordance with the modified version of Chen et al.’s methodology [31]. A total of 200 mg of KBr was combined with 1 mg of the sample in a 1:200 (*w*/*w*) ratio. Using 32 scans with a resolution of 4 cm^−1^, spectra between 400 and 4000 cm^−1^ were obtained in the absorbance mode.

### 2.9. Statistical Analysis

Each experiment was carried out three times. The analysis of variance (ANOVA) method was used to determine the means and standard deviations (SDs) of experimental data. Duncan’s comparison tests were performed to examine the outcomes using SPSS Statistics 23. The threshold for significance was *p* < 0.05.

## 3. Results and Discussion

### 3.1. Stability of Preheating WPC-ANs

#### 3.1.1. Storage Stability

**Figure 1:**
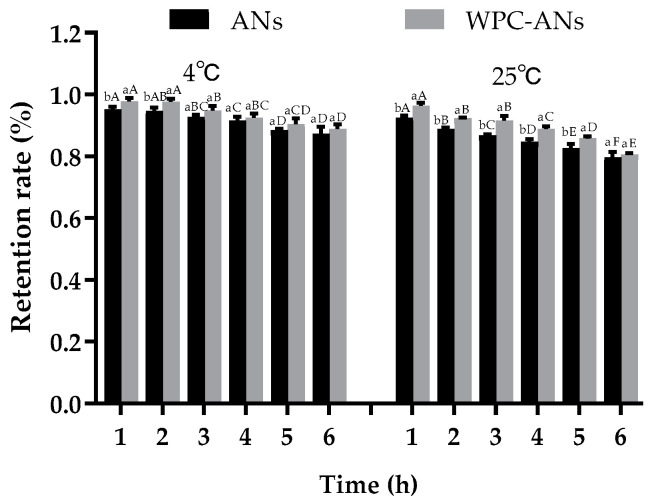
The retention rate of ANs at 4 °C and 25 °C after 6 h of storage, both with and without preheating WPC. Lowercase letters indicate significant differences between ANs and preheating WPC-ANs. Capital letters signify significant differences in time.

The effects of preheating WPC on the retention rate of purple sweet potato ANs were illustrated in Figure 1 over the course of the six-hour storage experiment. Due to ANs’ deterioration, the retention rate for all samples fell with time. After storage at 25 °C for 5 h, the retention rate of WPC-ANs (85.88%) was substantially higher than that of controls (82.65%, *p* < 0.05). This was attributed to the dense network structures of the gels generated from concentrated protein that encouraged the retention rate of ANs [32].

#### 3.1.2. Thermal Stability

**Figure 2:**
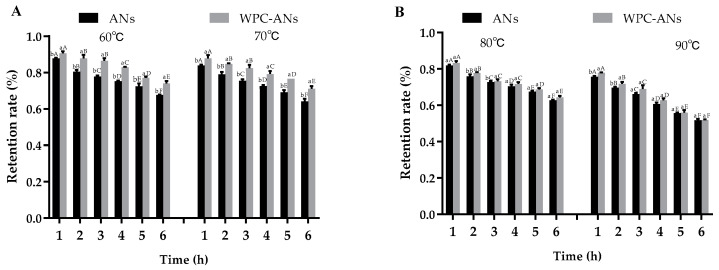
The retention rate of ANs at 60 °C (**A**), 70 °C (**A**), 80 °C (**B**), and 90 °C (**B**) after 6 h of heating, both with and without preheating WPC. Lowercase letters indicate significant differences between ANs and preheating WPC-ANs. Capital letters signify significant differences in time.

The food industry frequently uses thermal processing as a unit operation to preserve food safety and extend acceptable shelf life. More ANs degradation was caused by the longer heating time and higher temperature [33]. It was discovered that when heating temperature and time increased, the retention rate of ANs decreased dramatically for all samples (Figure 2A,B). Heated at 60 °C and 70 °C for 6 h, at 80 °C for 3 h, and 90 °C for 2 h, the addition of WPC greatly increased the retention rate of ANs. The retention of ANs in the complex after two hours of heating at 80 °C was 77.77%, which was higher than the protection provided by β-lactoglobulin for malvidin-3-O-glucoside (60.78–62.00%) under the same circumstances. At preheating temperatures, WPC would have more unfolded structures, making it easier for ANs molecules to enter the calyx structure [34]. ANs would be protected and stay out of the heat. This characteristic of preheating WPC-ANs gives them an advantage over ANs when used as food additives.

#### 3.1.3. Oxidation Stability

**Figure 3:**
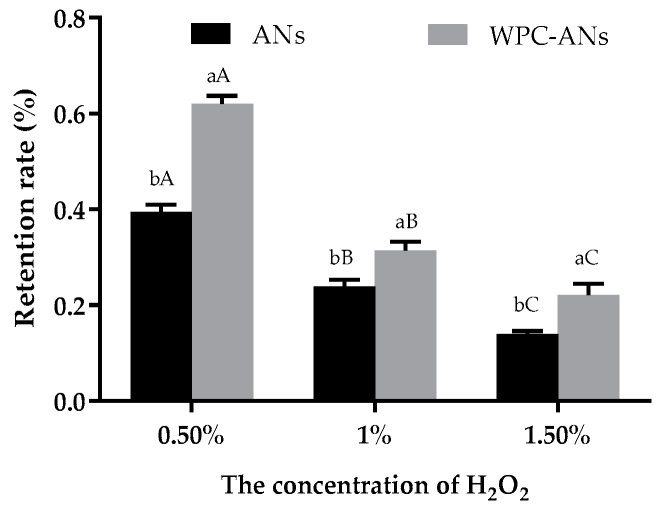
The retention rate of ANs by adding H_2_O_2_ (0.5%, 1%, and 1.5%), both with and without preheating WPC. Lowercase letters indicate significant differences between ANs and preheating WPC-ANs. Capital letters signify significant differences in the concenlration of H_2_O_2_.

As shown in Figure 3, when the concentrations of H_2_O_2_ were 0.50%, 1.00%, and 1.50%, the retention rate of ANs in the complexes was 62.04%, 31.43%, and 22.15%, respectively. They were noticeably higher than the control group’s (39.53%, 23.90%, 14.02%, *p* < 0.05). These findings were consistent with earlier research [19]. The retention rate of ANs of the heated soybean isolate-anthocyanin complex was only 54.60% when the H_2_O_2_ concentration was 0.01%. According to reports, WPC successfully maintained the bioactivity of ANs and increased their bioavailability because of the potent molecular interactions that β-lactoglobulin has with its ligands [30,31]. As a result, it is assumed that the protective impact of WPC on anthocyanins is also probably connected to the whey protein–anthocyanin complexations [31].

#### 3.1.4. Photo Stability

**Figure 4:**
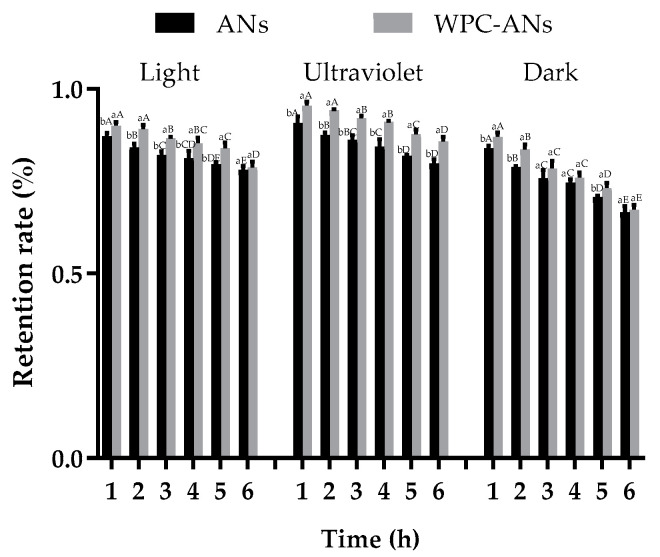
The retention rate of ANs kept for 6 h each in a light, UV lamp, and dark atmosphere, both with and without preheating WPC. Lowercase letters indicate significant differences between ANs and preheating WPC-ANs. Capital letters signify significant differences in time.

The retention rate of ANs considerably decreased with increased light time, as seen in Figure 4 (*p* < 0.05). This was due to the fact that prolonged exposure to light caused the carbon skeleton of ANs to break at the C_2_ position, forming intermediates of C_4_ hydroxyl. Then it was oxidized to chalcone. Chalcone underwent additional oxidation to produce ultimate hydrolysis products, including benzoic acid and 2,4,6-trihydroxybenzaldehyde, which caused anthocyanin to degrade and the color to fade [35]. The retention rate of ANs in WPC-ANs exposed to light and UV lamp for 6 h was 78.72% and 85.76%, respectively, which were significantly higher than the control (78.14% and 79.82%, *p* < 0.05). The non-covalent bonding between WPC and ANs, which enabled ANs to be effectively protected by WPC and resulted in increased bioavailability, was responsible for this [16].

#### 3.1.5. The Stability of WPC-ANs during Simulated In Vitro Digestion

**Figure 5:**
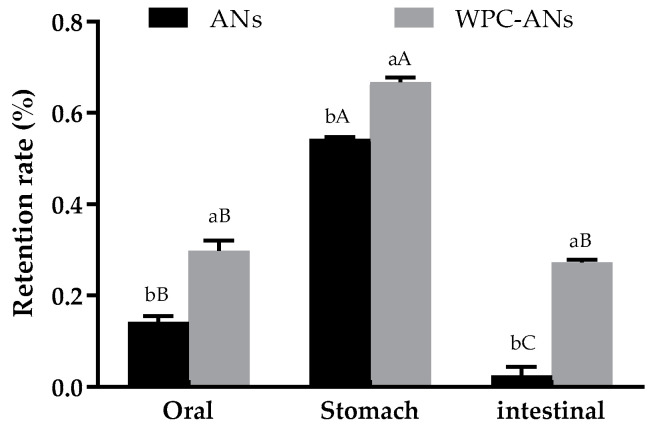
The retention rate of ANs in gastrointestinal simulation, both with and without preheating WPC. Lowercase letters indicate significant differences between ANs and preheating WPC-ANs. Capital letters signify significant differences in different digestive juices.

Figure 5 showed that, under the simulated salivary digestion, ANs degraded, most likely as a result of the mouth cavity’s digestive enzymes. After gastric digestion, the anthocyanin retention rates significantly increased, most likely because the bound form of ANs was changed to the free form under the effect of digestive enzymes and other factors [36]. The retention rate of ANs in simulated intestinal digestive juices was only 2.50%, which was likely due to the alkaline environment of intestinal fluid, which caused ANs to degrade and had limited accessibility [37]. In oral, gastric, and intestinal digestive juices, the retention rate of ANs in preheating WPC-ANs was 29.86%, 66.80%, and 27.27%, respectively, which were significantly higher than the control (14.26%, 54.29%, and 2.50%, *p* < 0.05). It was suggested that WPC had a beneficial protective effect on ANs in simulated digestive juices. This was explained by the fact that the interaction between the hydroxyl group of anthocyanins and the carbonyl (C=O), amino (-NH_2_), and hydroxyl (-OH) groups of polypeptides prevented the breakdown of ANs during simulated in vitro digestion [16].

### 3.2. Antioxidant Activity of WPC-ANs Mixtures during Simulated In Vitro Digestion

**Figure 6:**
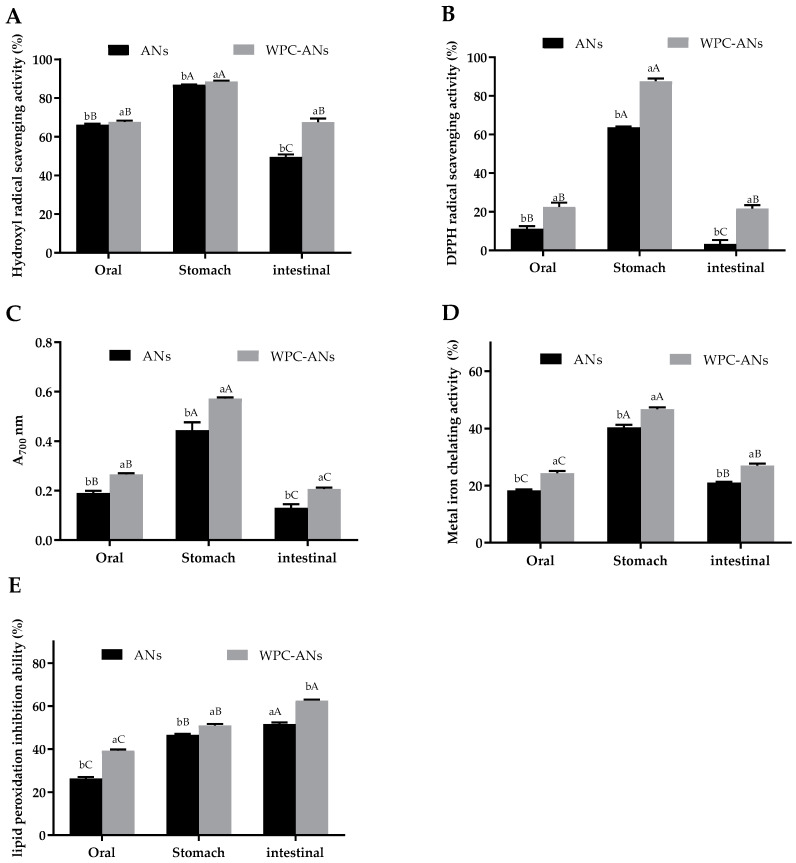
Antioxidant activity in gastrointestinal simulation, both with and without preheating WPC: hydroxyl radical clearance (**A**), DPPH radical clearance (**B**), chelating rate (**C**), metal iron chelating activity (**D**), and lipid hydrogen peroxide inhibition rate (**E**). Lowercase letters indicate significant differences between ANs and preheating WPC-ANs. Capital letters signify significant differences in different digestive juices.

To evaluate the antioxidant activity, reducing power, hydroxyl radical scavenging activity, 1,1-diphenyl-2-picrylhydrazyl (DPPH) tests, metal iron chelating activity, and lipid peroxidation inhibition ability were used in this study. Figure 6A,E show that each index of antioxidant activity of the complex was significantly higher than that of the control at each stage of digestion (*p* < 0.05). In oral, gastric, and intestinal digestive juices, the hydroxyl radical clearance of WPC-ANs was 67.73%, 88.66%, and 67.67%, respectively, which was significantly higher than the control (66.17%, 86.94%, 49.58%, *p* < 0.05). DPPH radical clearance of WPC-ANs was 22.52%, 87.62%, and 21.71%, respectively, which was significantly higher than the control (11.19%, 63.67%, 3.38%, *p* < 0.05). The chelating rate of WPC-Ans was 0.27, 0.57, and 0.21, respectively, which was significantly higher than the control (0.19, 0.45, 0.13, *p* < 0.05). The metal iron chelating activity of WPC-ANs was 24.37%, 46.68%, and 27.04%, respectively, which was significantly higher than the control (18.33%, 40.38%, 27.04%, *p* < 0.05). The lipid hydrogen peroxide inhibition rate of WPC-ANs was 35.32%, 70.03%, and 34.28%, respectively, which was significantly higher than the control (28.70%, 58.88%, 21.78%, *p* < 0.05). These findings implied that WPC greatly safeguarded ANs’ ability to act as antioxidants. Preheating WPC-ANs has better gastrointestinal bioaccessibility and antioxidant activity, which is expected to be used in the pharmaceutical industry.

### 3.3. Fluorescence Quenching of Preheating WPC-ANs Complexation

A protein’s tryptophan residues have inherent fluorescence, which can be utilized to study the interactions and binding of ANs with proteins [38]. Changes in emission peaks can be used to infer changes in protein structure and the physical and chemical environment around fluorophores. At the excitation wavelength of 280 nm, tryptophan (Trp) and tyrosine (Tyr) residues are both responsible for the fluorescence emission from proteins. However, at the emission wavelength of 295 nm, only Trp residues are responsible [39].

**Figure 7:**
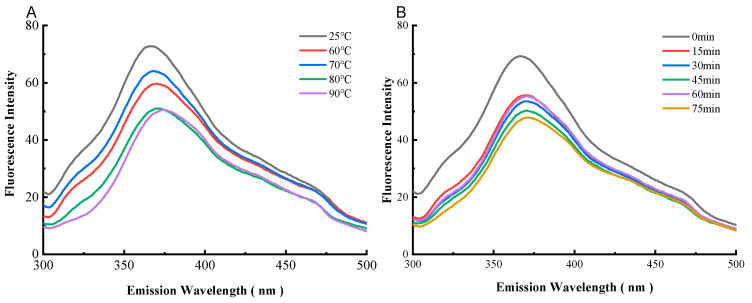
Fluorescence spectra of WPC heated for 60 min at different temperatures (25, 60, 70, 80, and 90 °C) in the presence of ANs with an excitation wavelength of 280 nm (**A**). Fluorescence spectra of WPC heated at 80 °C in the presence of ANs for different times (0, 15, 30, 45, 60, and 75 min) with an excitation wavelength of 280 nm (**B**).

The correlation between the fluorescence spectra and the WPC’s preheating temperature was shown in Figure 7A. The strength of the fluorescence signal from WPC-ANs reduced as the preheating temperature rose, and the maximum emission wavelength of WPC changed from 366.0 nm to 370.4 nm, 368.8 nm, 370.4 nm, and 373.6 nm (red shift) in that order. The correlation between the fluorescence spectra and the WPC’s preheating time was shown in Figure 7B. The strength of the fluorescence signal from WPC-ANs reduced as preheating time increased, and the λmax of WPC shifted toward the red, from 366.2 nm to 370.6 nm, 371.0 nm, 370.8 nm, 370.4 nm, and 371.6 nm, respectively. The red-shift proved that the microenvironment around the Tyr residues in WPC became more polarized. The endogenous fluorescence quenching of WPC by ANs was responsible for the decrease in fluorescence intensity [40].

**Figure 8:**
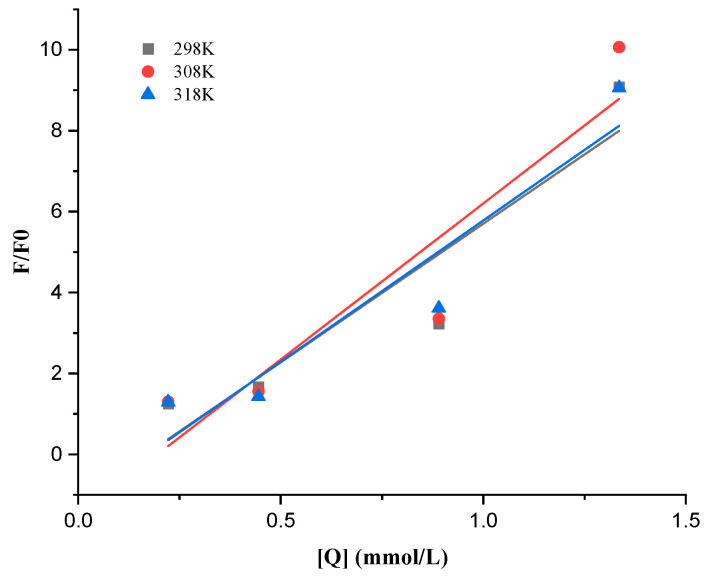
Stern–Volmer plots of WPC with ANs at 298, 308, and 318 K.

Small-molecule fluorescence quenching on proteins often involves both a dynamic and static quenching mechanism. Static quenching happens when a complex between the fluorophore and the quencher forms, whereas dynamic quenching depends on diffusion and collision encounters. The two quenching mechanisms have various temperature preferences. The quenching constant for dynamic quenching rises with temperature because more diffusion and collisions occur at higher temperatures. The complex formation, which regulates static quenching, could become less stable as a result of rising temperatures, which would then lead to a fall in the static quenching constant [41,42]. Figure 8 and Table 1 show the quenching parameters calculated from Equation (2), as well as the Stern–Volmer plot of WPC fluorescence quenched by ANs at 298, 308, and 318 K.

With rising temperatures, the *K_SV_* values of WPC were 6.844, 7.707, and 6.977 ×10^3^ L/mol, respectively. The *K_q_* values were substantially greater than the maximum dynamic quenching constant (2.0 × 10^10^/M·s), showing that purple sweet potato anthocyanins might reduce the fluorescence of the WPC by way of the static quenching brought on mostly by the formation of complexes [43].

**Figure 9:**
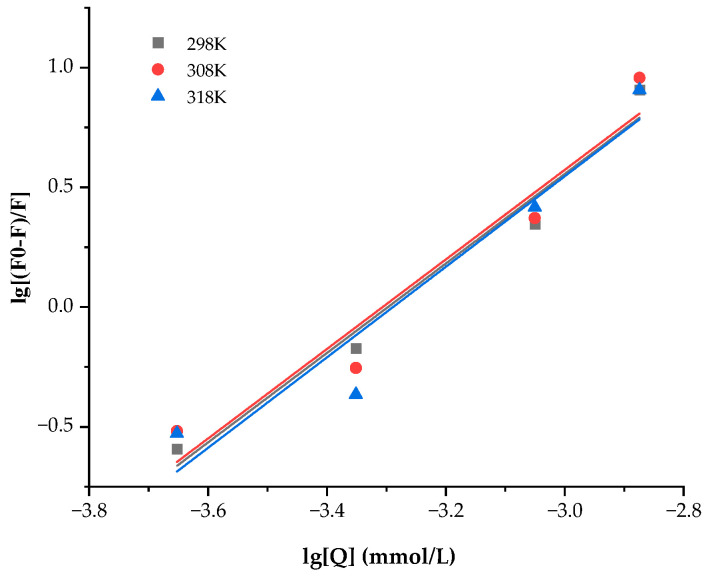
Double logarithmic regression plots of WPC with added ANs at 298, 308, and 318 K.

The double logarithm regression curves of log [(F_0_-F)/F] based on Equation (4) were depicted in Figure 9 for the static quenching process, and the *K_S_* and n were provided in Table 2 for the process. The *K_a_* between ANs and WPC was on the order of 10^6^ and rose as the temperature rose, showing that ANs had a high affinity for WPC and that their binding reaction was endothermic [18]. The n values were close to 1, suggesting that WPC only has one binding site in each AN during their interactions.

### 3.4. Thermodynamic Analysis When Preheating WPC Interacts with ANs

The analysis of thermodynamic parameters can be used to determine the types of non-covalent driving forces that are acting between a bioactive material and a protein. The binding force determines whether the thermodynamic parameters are positive or negative. The following connections exist: ΔH > 0 and S > 0 denoted hydrophobic interactions, ΔH < 0 and ΔS < 0 denoted van der Waals forces or hydrogen bonds, and ΔH < 0 and ΔS > 0 denoted electrostatic interactions. The positive values of ΔH and ΔS in Table 3 showed that interactions between ANs and WPC were primarily hydrophobic. Additionally, since ΔG was shown to be negative, ANs and WPC interacted spontaneously.

### 3.5. UV-Visible Absorption Spectroscopy of Preheating WPC-ANs

**Figure 10:**
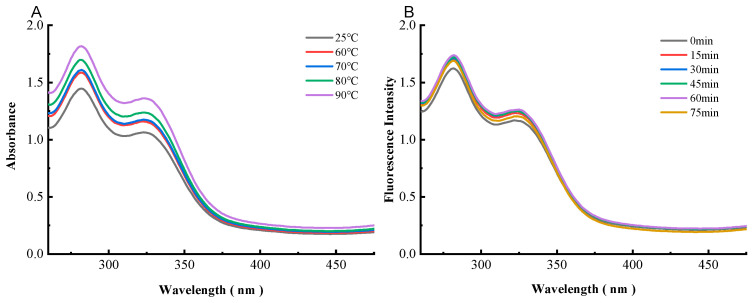
UV–visible absorption spectra of WPC heated for 60 min at different temperatures (25, 60, 70, 80, and 90 °C) in the presence of ANs (**A**). UV–visible absorption spectra of WPC heated at 80 °C in the presence of ANs for different times (0, 15, 30, 45, 60, and 75 min) (**B**).

The Trp and Tyr residues’ benzene heterocyclic rings undergo a transition known as a π → π* transition, which results in a shift in WPC’s UV-visible absorbance at 280 nm that provides information on the protein’s aromatic amino acid residues [15]. The impact of the preheating temperature of WPC on anthocyanins’ UV-visible spectra at 260–480 nm is depicted in Figure 10A. The intensity increase had changed after being preheating at different temperatures. The intensity sequence in this investigation was 90 °C > 80 °C > 70 °C > 60 °C > 25 °C, which might be because hidden hydrophobic groups were exposed or because different temperatures had an impact on secondary structural alteration. The influence of the preheating time of WPC on anthocyanins’ UV-visible spectra at 280 nm was depicted in Figure 10B. According to the aforementioned findings, the micro environment and secondary structure of amino acid residues may be affected by the preheating temperature and time. In other words, the UV-visible spectrum provided more evidence that the structure of WPC was altered by anthocyanins.

### 3.6. FTIR of Preheating WPC-ANs

The secondary structural alterations in proteins caused by the addition of anthocyanin can be assessed using FTIR [44]. Protein secondary structure is connected with both intensity shifts and spectral shifts of the amide I band, which is primarily C=O stretched at 1600–1700 cm^–1^, and the amide II band, which is C–N stretched and N–H bent at 1548 cm^–1^. The amide I band is more responsive to changes in the secondary structure of proteins than the amide II band is [45].

**Figure 11:**
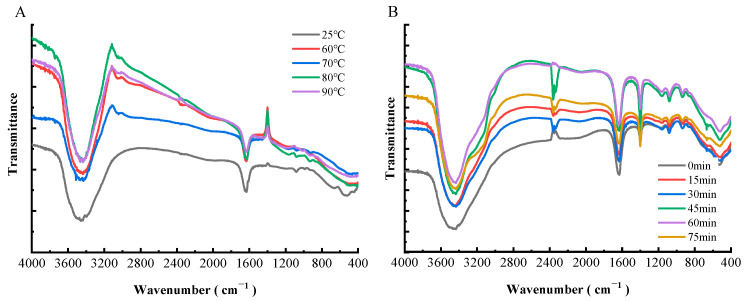
FTIR spectroscopy of WPC heated for 60 min at different temperatures (25, 60, 70, 80, and 90 °C) in the presence of ANs (**A**). FTIR spectroscopy of WPC heated at 80 °C in the presence of ANs for different times (0, 15, 30, 45, 60, and 75 min) (**B**).

As can be seen from Figure 11A, the amide I band showed a red shift from 1631.5 cm^−1^ (25 °C) to 1633.4 cm^−1^ (60, 70, and 80 °C), and finally 1637.3 cm^−1^ (90 °C), with an increase in preheating temperature. Both amides I and II lost some of their intensity. As demonstrated in Figure 11B, the amide I band of WPC-ANs complexes displayed a red shift from 1631.5 cm^−1^ to 1633.4 cm^−1^, 1637.3 cm^−1^, and 1633.4 cm^−1^, and the amide II band was blue-shifted from 1402 to 1400.1 cm^−1^, with an increase in preheating time. Additionally, the amide I and II bands’ intensity was reduced when the preheating time was extended. Red-shift and blue-shift phenomena showed how the secondary structure of WPC will alter to variable degrees depending on the preheating temperature and preheating time [45]. The amide I and II bands’ diminished intensity of WPC further suggested a decline in the amount of α-helix in WPC [45]. Additionally, the oxygen atom and hydroxyl group on ANs might bind to the C=O and C-N groups of WPC by hydrophobic contact, causing a reorganization of the carbonyl hydrogen bonding network of WPC peptides and reducing the intensity [46]. The FTIR results further demonstrated that various preheating temperatures and times resulted in various structural alterations in WPC and might provide further stability to the anthocyanins.

## 4. Conclusions

The obtained findings explicitly indicate that ANs’ stability and antioxidant activity were improved by preheating WPC. Preheating WPC’s non-covalent attachment to ANs benefited ANs by providing protection. The WPC-ANs connection was static quenching, and the binding force between them was a hydrophobic interaction at one of the binding sites, according to the fluorescence quenching spectroscopy. The results of UV-visible spectroscopy and Fourier transform infrared spectroscopy (FTIR) analysis further indicated that the secondary structure and microenvironment of amino acid residues in WPC can be impacted by the preheating temperature and preheating duration of WPC. All of the results showed that preheating encouraged WPC binding to ANs and further safeguarded their stability. This study offered theoretical justification for the use of purple sweet potato preheating WPC-ANs in the food industry and pharmaceutical industry.

## Figures and Tables

**Table 1:** Stern–Volmer quenching constant of preheating WPC-ANs at 298, 308, and 318 K.

Sample	Temperature/K	*K_sv_*/(×10^3^ L/mol)	*K_q_*/(×10^11^ L/(mol·s))	R^2^
WPC-ANs	298	6.844	6.844	0.8732
	308	7.707	7.707	0.8618
	318	6.977	6.977	0.9003

**Table 2:** Binding constants, binding sites, and linear correlation coefficients for the interaction of WPC with ANs at 298, 308, and 318 K.

Sample	Temperature/K	*K_a_*/(×10^6^ L/mol)	*n*	R^2^
WPC-ANs	298	1.449	1.8683	0.9710
	308	1.522	1.8697	0.9395
	318	1.652	1.8905	0.9246

**Table 3:** Thermodynamic parameters, at various temperatures (298 K, 308 K, and 318 K) for the preheating WPC-ANs.

Sample	Temperature/K	ΔH/(kJ/mol)	ΔG/(kJ/mol)	ΔS/(kJ/mol)
WPC-ANs	298	5.164	−35.147	135.272
	308		−36.454	135.121
	318		−37.854	135.275

## Data Availability

The data used for the research described in this manuscript are available upon request.
